# Epilepsy Mechanisms in Down Syndrome: Genetic and Pathophysiological Perspectives Across the Lifespan

**DOI:** 10.3390/jcm15197471

**Published:** 2026-09-25

**Authors:** Carl E. Stafstrom, Li-Rong Shao

**Affiliations:** Division of Pediatric Neurology, Department of Neurology, Johns Hopkins University School of Medicine, Baltimore, MD 21287, USA; lshao5@jhmi.edu

**Keywords:** infantile epileptic spasms syndrome (IESS), Down syndrome, late-onset myoclonic epilepsy in Down syndrome (LOMEDS), Alzheimer disease, dementia

## Abstract

Down syndrome (trisomy 21) is the most common genetic cause of intellectual disability. Seizures are a frequent comorbidity in individuals with Down syndrome and can exert significant negative consequences on cognitive capacity and quality of life. In Down syndrome, seizures cluster at two extremes of the lifespan. In the first year of life, infantile epileptic spasms syndrome affects 5–10% of children with Down syndrome. The second peak occurs after age 30 years, when myoclonic seizures appear at the time of onset of Down syndrome-related dementia of the Alzheimer type (late-onset myoclonic epilepsy in Down syndrome or LOMEDS). While epilepsy at these two disparate time points has been long recognized in the Down syndrome population, the mechanisms underlying seizure pathogenesis have not been studied in detail. For example, it is not known whether the brain developmental abnormalities that characterize Down syndrome are primarily responsible for the epilepsies or whether distinct mechanisms underlie seizures at the two ages. This narrative review addresses possible mechanisms of seizure propensity across the lifespan, with special reference to potential age-specific treatments.

## 1. Introduction

Down syndrome (trisomy 21), the most common genetic cause of intellectual disability, is a well-recognized genetic disorder caused by triplication of human chromosome 21 (Hsa21) [1,2]. Down syndrome has pleiotropic manifestations involving many body systems, including the heart, immune system, gastrointestinal system, and, most notably, the brain. Neurological manifestations of Down syndrome include decreased intellectual function, hypotonia, and seizures [3]. In Down syndrome, seizures have a bimodal age distribution, with about 40% of cases beginning in the first year of life and another 40% starting in adulthood [4,5]. Figure 1 shows a graph of approximate seizure incidence in Down syndrome as a function of age, indicating these two peaks. The early peak is most often due to infantile epileptic spasms syndrome (IESS), whereas the peak later in life is related to the early onset of dementia in this population, leading to the unique epilepsy syndrome known as late-onset myoclonic epilepsy in Down syndrome (LOMEDS) [6]. Overall, between 1 and 13% of individuals with Down syndrome develop epilepsy [7]. Reasons for the increased prevalence of seizures in children with Down syndrome relate to two major factors: first, structural abnormalities of brain development and, second, medical complications of the syndrome (e.g., congenital heart and cardiovascular disease [8] including Moyamoya disease [9], increased incidence of infections, immune dysfunction) [10]. After about 30 years of age, when most individuals with Down syndrome develop dementia, there is a concomitant increase in seizures probably related to similar pathophysiological reasons as in individuals with Alzheimer’s disease [11,12,13].

This review explores the clinical features and pathophysiological underpinnings of seizures at these two extremes of the lifespan, with the recognition that Down syndrome is both a neurodevelopmental and neurodegenerative disorder [14]. Our focus in this review is on epileptogenic mechanisms, with clinical features included for perspective (Table 1) and to guide basic science investigations.

Neuropathology in Down syndrome is best envisioned as a continuum, whereby early developmental abnormalities also affect later brain function and dysfunction [15]. Trisomy of chromosome 21 affects brain development as early as prenatally, with altered neurogenesis (neurons and glia) [16,17], synaptogenesis, myelination, and many other processes that vary over the lifespan (e.g., oligodendrocytes, astrocytes), inflammation, or metabolic derangement [18,19]. To date, all of these topics have been understudied in regard to seizure predisposition in Down syndrome. Newer genetic technologies are uncovering interactions of genes, synapses, and neuronal networks across the lifespan that will be informative about intellectual disability and dementia in Down syndrome and will also likely enhance our knowledge about seizure mechanisms. Specific clinical questions to be informed by basic investigations include the frequency of seizures in Down syndrome at different ages, what factors across the lifespan in the Down syndrome brain favor increased seizure predilection, whether special management approaches are indicated compared to the general population, and how seizures affect the prognosis and natural history of Down syndrome.

## 2. Methods

This review provides an overview of the mechanisms for seizures and epilepsy in individuals with Down syndrome across the lifespan. We provide clinical information as an overview and aim to avoid extrapolation of preliminary animal data to clinical practice. 

A literature search was performed using the PubMed/MEDLINE database to identify relevant publications available from database inception through to 1 August 2026. The search strategy combined Medical Subject Headings (MeSH) terms, including “Alzheimer disease AND seizures”, Alzheimer disease AND epilepsy (to identify seizure mechanisms in AD); dementia AND seizures, dementia AND epilepsy (to identify seizure mechanisms in dementia etiologies in non-Alzheimer cases); Down syndrome OR trisomy 21 and seizures OR epilepsy OR infantile spasms; and each of the above terms AND animal models.

Eligible publications included reviews, clinical trials, pharmacological studies, and studies involving relevant animal models and disease mechanisms. Editorial comments without original scientific content and conference abstracts were not considered.

As a narrative review, no formal systematic review methodology, PRISMA reporting framework, or quantitative assessment of study quality were used. Studies were evaluated for their scientific merit and impact, clinical relevance, and contribution to understanding the mechanisms by which seizures occur in this genetic disorder. No AI tools were utilized for the preparation of this review.

## 3. Why Are Seizures Increased in Down Syndrome?

A seizure is defined as a paroxysmal brain dysfunction resulting from abnormal neuronal firing, leading to increased network synchrony. The term epilepsy is applied when seizures occur without provocation, while an epilepsy syndrome entails similar seizure etiology, semiology, medication responsiveness, natural history, and other factors [20]. The presence of epilepsy implies that the brain circuits have become susceptible to spontaneous seizure generation.

In people with Down syndrome, abnormal brain development begins early in gestation and persists across the lifespan [15,21]. Pathological studies have demonstrated diminished brain growth from a macroscopic level with decreased brain volume, simplified sulcation, and a thinner cerebral cortex. Microscopically, there are fewer neurons, increased astrocytes, decreased axonal projections, and decreased oligodendrocytes with subsequent abnormal myelination. The presence of fewer dendritic arborizations and malformed dendritic spines in Down syndrome brains may underlie the decreased cognitive function and neuronal network activity [22]. Specifically, it has been proposed that inhibitory neural activity is relatively increased in the Down syndrome brain as an explanation for the cognitive slowing. That is, in Down syndrome, it has been hypothesized that the brain’s excitation/inhibition (E/I) balance is altered in favor of increased inhibition [23,24,25]. However, epilepsy is generally considered to reflect the opposite situation, with an increased E/I ratio reflecting a relative predominance of excitation over inhibition [26]. Therefore, increased inhibitory activity is opposite of what would be expected for epilepsy. How can this apparent contradiction be reconciled?

From a simplistic point of view, the molecular and pathophysiological alterations in Down syndrome might be hypothesized to result from triplication of genes located on chromosome 21. In trisomy 21, it has been demonstrated that numerous other genetic factors are also involved including effects of chromosome 21 alterations on genome-wide function [27,28]. Altered expression and regulation of many genes on Hsa21 and elsewhere contribute to the neuropathology and abnormal neuronal function in Down syndrome (Table 2). These abnormalities might include genes for excitability regulation involving synaptic function, brain development, and even ion channels; using induced pluripotent stem cells from Down syndrome individuals, excitatory dysfunction has been implicated in altered neuronal firing rate and network bursting, attributed to K^+^ and Na^+^ channel dysfunction, which could play a role in seizure genesis [29]. Other genes triplicated in trisomy 21 that might affect neuroexcitability include the transcription regulator DYRK1A and the cell adhesion molecule DSCAM, but neither has yet been shown to play a role in seizure propensity [30,31,32,33,34]. Furthermore, other neurotransmitter systems might be implicated in seizure propensity in Down syndrome. For example, in Down syndrome, degeneration of noradrenergic locus coeruleus neurons leads to neuroinflammation and blood–brain barrier disruption, comprising a potential target for epileptogenesis that remains to be explored [35,36].

To explore the structural and pathophysiological features of Down syndrome, numerous animal models have been developed, usually using mice [37,38,39]. Each model has a variable fidelity with human Down syndrome. It is acknowledged that no animal model perfectly replicates human Down syndrome, but the models can be used to ask specific mechanistic and translational questions. Other experimental systems, such as induced pluripotent stem cells, can provide ancillary information, but for a network disorder such as epilepsy, animal models are indispensable.

Two animal models are outlined here, chosen for discussion because of evidence for increased seizure predisposition. First, Ts65Dn is by far the most well-studied model, having been developed more than three decades ago [40]. This model is characterized by segmental trisomy of mouse chromosome 16, which harbors about 60% of the human chromosome 21 protein coding genes. Ts65Dn mice mimic many of the neuroanatomical and physiological abnormalities seen in human Down syndrome, including small overall brain growth, excess inhibitory interneurons, simplified dendritic arbors [41], and impaired synaptic plasticity, learning, and memory [42,43]. However, several genes are present in Ts65Dn mice that are not triplicated in human trisomy 21, and many genes seen in human Down syndrome are absent from Ts65Dn. Therefore, this model may have limited applicability to human Down syndrome [44].

The second model, TcMAC21, is created by genetic engineering of human chromosome 21 into the genome of a mouse through successive gene transfers [45]. TcMAC21 mice harbor 93% of orthologous Hsa21 protein coding genes. TcMAC21 mice are non-mosaic, unlike other models. The phenotype is less severe than Ts65Dn, but TcMAC21 mice are more similar genetically to human trisomy 21. TcMAC21 mice have enlarged ventricles and superior colliculus, a small cerebellum, modest learning the memory deficits in the water maze and open field, and altered motor coordination and vocalizations [45]. TcMAC21 mice have been shown to display disruption of excitatory cortical progenitor cells in mid gestation, leading to altered postnatal connectivity and electrical activity [46,47], but a direct relationship between those developmental changes and seizure predilection has not been obtained.

Of note, spontaneous seizures have not been reported in either Ts65Dn or TcMAC21 mice, though both models have a lower threshold to a variety of seizure-inducing stimuli (e.g., GABA_B_-R agonists) and exhibit pathophysiological differences that are useful for specific questions about seizure mechanism and potential therapeutic considerations. Furthermore, as discussed below, Ts65Dn and TcMAC21 mice have an increased predisposition to seizures (especially infantile epileptic spasms), making them attractive for study of developmental seizure mechanisms [48,49]. Eventually, other models may prove informative, e.g., Ts66Yah [50], but seizures have not been noted or studied other than Ts65Dn and TcMAC21.

## 4. Early Life Epilepsy in Down Syndrome: Infantile Epileptic Spasms Syndrome

Infantile spasms epileptic syndrome (IESS), previously called infantile spasms or West syndrome, is an age-dependent epileptic encephalopathy with onset in the middle of the first year of life [51]. IESS can result from more than 200 acquired or genetic etiologies [52,53]. The incidence of infantile spasms in Down syndrome is between 1 and 10% (probably closest to 5%), which is roughly 100-times the general population’s incidence of IESS [54,55]. The seizures in IESS involve flexion or extension spasms in clusters, and this semiology is crucial to understanding mechanisms by which spasms develop; while the detailed localization of spasms onset and propagation have not been elucidated, they likely involve interaction between cortical and subcortical (thalamic, brainstem) structures [51]. IESS is often accompanied by a slowing or regression of development, a key concern in Down syndrome individuals who are already limited cognitively [56,57]. The typical interictal EEG pattern, characterized by high-amplitude chaotic slow waves with interposed spikes, is known as hypsarrhythmia. Hypsarrhythmia in children with Down syndrome who develop IESS is qualitatively similar to that seen in the non-Down syndrome population [51,58]. When a seizure (spasm) occurs, all EEG waveforms briefly attenuate, called the electrodecremental response. The treatment options for IESS are different from most other epilepsy syndromes, with the most effective treatments being adrenocorticotrophic hormone (ACTH) or oral corticosteroids (OCS, prednisolone), both in Down syndrome and non-Down syndrome individuals [59,60,61,62]. Vigabatrin, a gamma-aminobutyric acid (GABA) reuptake inhibitor, has been shown to be effective for IESS due to tuberous sclerosis, and some authorities have advocated its use in other epilepsies such as Down syndrome, but it is less effective than ACTH or OCS [63,64,65]. The prognosis of IESS is variable, but the Down syndrome population is already at risk for cognitive dysfunction, and IESS could exacerbate that risk [56,57,59].

The search for the mechanism of IESS in Down syndrome has focused on animal models at three basic levels of investigation: seizure susceptibility (whole animal/network level), neuronal excitability (intrinsic membrane defects at single-cell level), and synaptic dysfunction [66,67]. The vulnerability of Ts65Dn mice to epileptic spasms was first demonstrated by administration of GABAB-R agonists, which led to spasms and accompanying EEG changes consisting of epileptiform bursts and electrodecrements [48]. These mice responded to two standard treatments of IESS, ACTH and vigabatrin, adding to the face validity of the model. However, it is not clear why GABAB-R agonism leads to spasms occurrence, because GABAB-R activation leads to increased inhibition: intracellular G-protein activation of GIRK2 (G-protein inward rectifying potassium channels, type 2). The GIRK2 protein is overexpressed in Ts65Dn mice due to triplication of its encoding gene, *Kcnj* [68,69]. When activated, the GIRK2 channel effluxes K^+^ from the inside of the neuron to the outside of the neuron, thereby hyperpolarizing the neuron. Neuronal hyperexcitability and seizure occurrence would not be predicted by K^+^ efflux. There are several possible alternative mechanisms by which GIRK2 overactivation leads to hyperexcitability, though none of them has supportive data at this time [70]. One alternative mechanism could involve GIRK2’s presynaptic action of blocking Ca^2+^ channels; this would reduce neurotransmitter release, but it is usually presumed that the transmitter in question whose release is reduced is glutamate, so that mechanism would also not explain increased seizure propensity. Another alternative is that GIRK2 overexpression might augment the hyperpolarization during GABAB-R activation of thalamic relay neurons, leading to enhancement of hyperpolarization-evoked “rebound bursts” in these neurons (a unique and well-known characteristic of thalamic neurons) and, thus, contribute to seizure propensity [71]. A third possibility is that GIRK2 inhibitory signaling in Ts65Dn hippocampal dentate gyrus neurons is increased, especially in discrete dendritic regions [72], an effect that can be reversed by a GABAB-R antagonist called CGP35348 [66]. A GABAB-R antagonist would be predicted to reduce inhibition, thus exacerbating excitation and provoking spasms. Developmental homeostatic plasticity of GABAergic neurotransmission might counter the increased GIRK2-mediated inhibitory signaling and restore E/I balance [73]. Explaining increased neuronal excitability in the setting of excess inhibition remains a critical translational challenge [74,75].

While the role of GABAB-R remains uncertain in Down syndrome, GABAA-R areis well positioned to account for seizure predisposition and cognitive status [76]. Early in life (until about term in humans and until the late second week of postnatal life in rodents [77]), GABA mediates depolarization and neuronal excitation rather than the mature situation in which GABAA mediates hyperpolarization. Thus, over development, GABA’s action transitions from depolarizing to hyperpolarizing and inhibitory [78]. If this transition is delayed, seizure susceptibility could persist over a longer age window. Using hippocampal cultures and brain slices, the GABA polarity switch was found to be delayed by 2-3 days in the Ts65Dn model [79,80]. Whether this delay is sufficient to affect susceptibility to spasms is not known [81]. Tools are becoming available to restore excessive GABAA signaling, including bumetanide (blocker of the NKCC1 chloride importer [82]) and RNA interference or viral-induced neuron-specific NKCC1 knockdown [83], and could be used to dissect seizure mechanisms.

We have begun to explore inhibitory GABA-R effects as well as excitatory synaptic mechanisms using the TcMAC21 mouse model, with the hypothesis that triplicated genes governing neuronal and synaptic excitability are involved in IESS. Three such genes that are located on chromosome 21 and, thus, could affect seizure generation are *GIRK2* (encodes GABA-B receptor, regulates tonic inhibitory neurotransmission), *GRIK1* (encodes kainate receptor, a major category of glutamate receptor), and *SYNJ1* (encodes synaptojanin, synaptic vesicle recycling and trafficking regulator [84]). Of course, numerous other genes are also likely to be involved in governing neuronal excitability (Table 2), but these three genes form a starting point for investigations of the mechanism, with the hope that a novel therapeutic target may emerge.

Seizure susceptibility at the whole animal level was studied in response to the GABA-B receptor agonist gamma-butyrolactone (GBL) and the GABA-A receptor antagonist, flurothyl. We found that GBL caused clusters of spasms in 85% of TcMAC21 mice and only 25% of euploid mice, a highly significant difference [85]. EEGs on TcMAC21 mice revealed bursts of spike wave activity during the clinical spasms. Similarly, young TcMAC21 mice (~2–3 weeks of age [86]) had shorter latencies to flurothyl-induced seizures than euploids, suggesting increased sensitivity to GABA-A receptor activation [87]. Therefore, both GABA-A and GABA-B receptor activities are altered in TcMAC21 mice. At a cellular level, membrane properties of euploid and TcMAC21 mice were investigated using depolarizing and hyperpolarizing current pulses in current-clamped neocortical-layer 5 neurons, which are likely involved in epileptic spasms generation [88]. No differences were found in the ability of neurons of each group to fire repetitively or in membrane properties (input resistance, action potential threshold, rheobase, firing rate) (Figure 2A), though a recent report using induced-pluripotent stem cells showed decreased neuronal firing rate and reduced network bursting, implicating abnormal voltage-gated sodium and potassium channels [29]. Therefore, further investigation of TcMAC21 neurons at the single-cell level could be informative.

At the synaptic level, however, there were marked differences between euploid and TcMAC21 neocortical neurons. Excitatory postsynaptic currents were significantly larger in TcMAC21 neurons than in euploid neurons, resulting in an increased E/I ratio in the TcMAC21 model [85]. We further demonstrated that TcMAC21 neurons were more sensitive to kainate, a glutamate receptor analog [89] (Figure 2B). These findings are consistent with overexpression of the *GRIK1* gene for kainate receptors (KA-R), found on Hsa21. We also found that during high-frequency synaptic activity, neurotransmitters are less readily depleted in TcMAC21 neurons than in euploid neurons [89], possibly related to increased *SYNJ1* expression, which might also support epileptic firing.

A caveat when considering excitation versus inhibition in infantile spasms pathophysiology is the neuron type studied. It is not certain which areas of the brain are responsible for or involved in spasms generation, let alone the situation in Down syndrome and its models. Most studies using the Ts65Dn mouse have investigated the hippocampus [42,79], which is clearly critical for learning and memory but is not usually implicated in epileptic spasms. Only a few reports have studied neocortical neurons [85,87,89,90], and a critical gap is elucidation of the role of the thalamus and brainstem circuit in IESS, in Down syndrome and other etiologies [48,88]. There may exist age- and regional-specific differences in physiological consequences of overexpression of Down syndrome genes.

In summary, investigations to date using the Ts65Dn and TcMAC21 mouse models of Down syndrome have provided intriguing preliminary results. Mice brains from both models are highly prone to clinical epileptic spasms, particularly when GIRK2 channels are activated by GABA-B receptor agonists. Ts65Dn mice demonstrate a prolonged GABA-A depolarizing action, while TcMAC21 mice exhibit an increased E/I ratio due to increased sensitivity to kainate and dysfunction of neurotransmitter recycling [49,78,81,88] (Figure 3 and Figure 4A). Other mechanisms also likely play a role, but the factors in Figure 3 are based on published reports. Factors such as inflammation, circuit-level and regional heterogeneity, and age correlations require elucidation. Pharmacological trials for spasms are a critical priority. So far, IESS in Down syndrome seems to respond to the same standard medications that are effective in other etiologies, that is, ACTH and OCS.

## 5. Later-Life Epilepsy in Down Syndrome: LOMEDS

With improved medical care, more individuals with Down syndrome are living to older ages. After age 30, individuals with Down syndrome develop a form of dementia consistent with early Alzheimer disease [91,92]. In fact, Down syndrome is now considered to be the most prevalent genetic form of Alzheimer’s disease. Concurrent with the recognition of this early dementia, it was noted that older people with Down syndrome developed a unique kind of epilepsy involving myoclonic seizures (with or without generalized tonic–clonic seizures). This form of myoclonic epilepsy is considered to be progressive in that its prevalence and effects accumulate with time, causing diminished quality of life in affected individuals [6,93,94,95]. At least 50% of individuals with Down syndrome that develop Alzheimer’s dementia will develop LOMEDS [96]. EEGs have shown diffusely slow backgrounds with generalized polyspike wave bursts during the myoclonic seizures [97,98]. The appearance of LOMEDS is highly correlated with rapid cognitive decline and dementia [96,99,100]. Furthermore, the long-term prognosis in Down syndrome patients who develop myoclonic epilepsy is dire. D’Orsi and colleagues recently summarized the 11-year follow-up in a cohort of 12 patients with LOMEDS originally identified in 2014 [95,101]. Median survival after myoclonic epilepsy onset was only about 4 years, with a steady decline in cognitive function and quality of life in those terminal years [101]. The authors emphasize the progressive nature of functional deterioration once seizures begin, reflected in their proposed renaming of the syndrome as Progressive Myoclonus Epilepsy–Down syndrome (PME-DS).

The occurrence of neuronal hyperexcitability in the form of epilepsy is well recognized in the Alzheimer’s disease population [11,12,13,102,103,104]. Both amyloid beta (Aß) protein and tau protein deposition lead to neuronal hyperexcitability and alter the E/I balance, and clinically, the effects of each might be differentiable using techniques such as magnetoencephalography [105].

The gene for Aß protein (*APP*, amyloid precursor protein) is found on chromosome 21, so its gene dosage effect contributes to the neuropathological manifestations of early dementia. In animal models, Aß accumulation leads to neural and synaptic degeneration, neuroinflammation, increased neuronal excitability, and epilepsy [106,107,108]. Recently reported mouse models incorporating Aß humanization may allow for earlier identification of pathological mechanisms and, hence, treatment options [108]. Seizures increase Aß release and seizures exacerbate neuronal damage, leading to a vicious cycle of pathophysiology [11,104,109,110,111] (Figure 4B). In mutant mice overexpressing APP, there are epileptiform discharges and overt and subclinical seizures, as well as increased activity in inhibitory circuits [106]. In addition, tau protein, typically involved in neurofibrillary tangles, also alters neuronal excitability, increasing extracellular glutamate and leading to N-methyl-D-aspartate (NMDA) receptor dysfunction. In Alzheimer’s disease, tau is hyperphosphorylated, resulting in reduced tau binding to microtubules and the formation of neurofibrillary tangles [107,111]. Extrapolating from Alzheimer’s disease to Down syndrome, it is likely that similar mechanisms underlie the heightened excitability and seizures in LOMEDS. In humans, positron emission tomography (PET) imaging demonstrated marked accumulation of Aß in Down syndrome individuals who developed myoclonus and dementia [101]. Numerous opportunities to ameliorate abnormal Aß and tau dysfunction hold promise for similar interventions in LOMEDS [112,113]. Chromosome 21 genes other than *APP* also affect learning and memory, either positively or negatively. For example, interferon (IFN) upregulation in Down syndrome animal models may represent a target for cognitive remediation [114] and also plays a role in excitability regulation through the JAK-STAT pathway [115,116]. There is increasing interest in using Down syndrome models such as Ts65Dn to explore mechanisms of neurodegeneration and seizure susceptibility [107,111,117]. The TcMAC21 mouse also expresses increased levels of APP and Aß but no plaque disposition at 24 months of age, although cognitive deficits are demonstrated far earlier [45,107]. Therefore, both models have the potential to explore the overlap between cognitive impairment and increased seizure predisposition.

It is unknown why seizures in LOMEDS tend to be myoclonic in type. In Alzheimer’s disease, myoclonic seizures often arise from a cortical site, whereas in LOMEDS, an EEG correlate is often lacking in the later stages of the disorder, possibly indicating a subcortical generator [96]. A critical unresolved question is whether epileptic activity (subclinical or overt) is clinically significant in the dementia of Down syndrome and whether early treatment would benefit patients [118]. For LOMEDS, anticholinesterases, tau-directed agents, mTor inhibitors, and others warrant study beyond the conventional anti-seizure medications typically used.

In summary, mouse models of Alzheimer’s disease provide an analog for the development of dementia and myoclonic seizures in Down syndrome. Aß and tau facilitate increased neuronal excitation and seizures and accelerate cognitive decline. Treatment of the myoclonic seizures of LOMEDS is a critical clinical challenge because of the dire prognosis and imminent mortality involved.

## 6. Conclusions and Future Directions

Down syndrome is clearly both a neurodevelopmental and neurodegenerative disorder with manifestations across the lifespan, beginning prenatally and extending into adulthood. Epilepsy manifestations and consequences comprise essential clinical components mirrored over the lifespan. Future investigations will focus on further elucidation of the pathophysiology of both IESS and LOMEDS, the roles of chromosome 21 genes and others on network excitability, the extent of GABA’s depolarizing action on seizure predisposition, and the involvement of thalamocortical circuits. These and other factors will inform potential targets for seizure abatement in Down syndrome at multiple stages of the lifespan. Finally, it is critical that any relationship between early life IESS and later LOMEDS be elucidated.

## Figures and Tables

**Figure 1:**
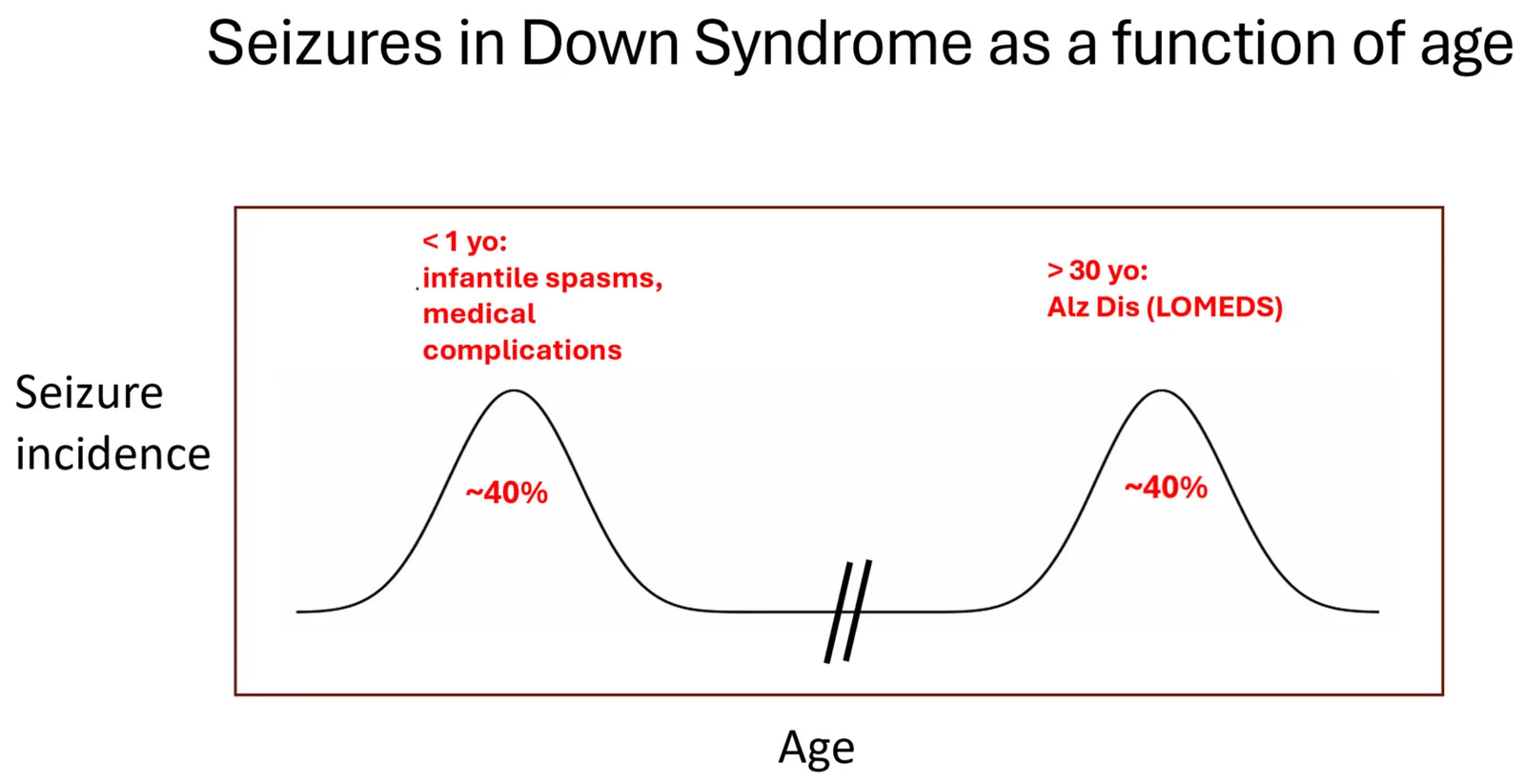
Seizure incidence in Down syndrome as a function of age. Two peaks are evident, during the first year of life when infantile spasms predominate, and after age 30 when myoclonic epilepsy ensues as a consequence of Alzheimer’s disease. Data derived from [4]. Notes: yo, years old; Alz Dis, Alzheimer’s disease; LOMEDS, late-onset myoclonic epilepsy in Down syndrome.

**Figure 2:**
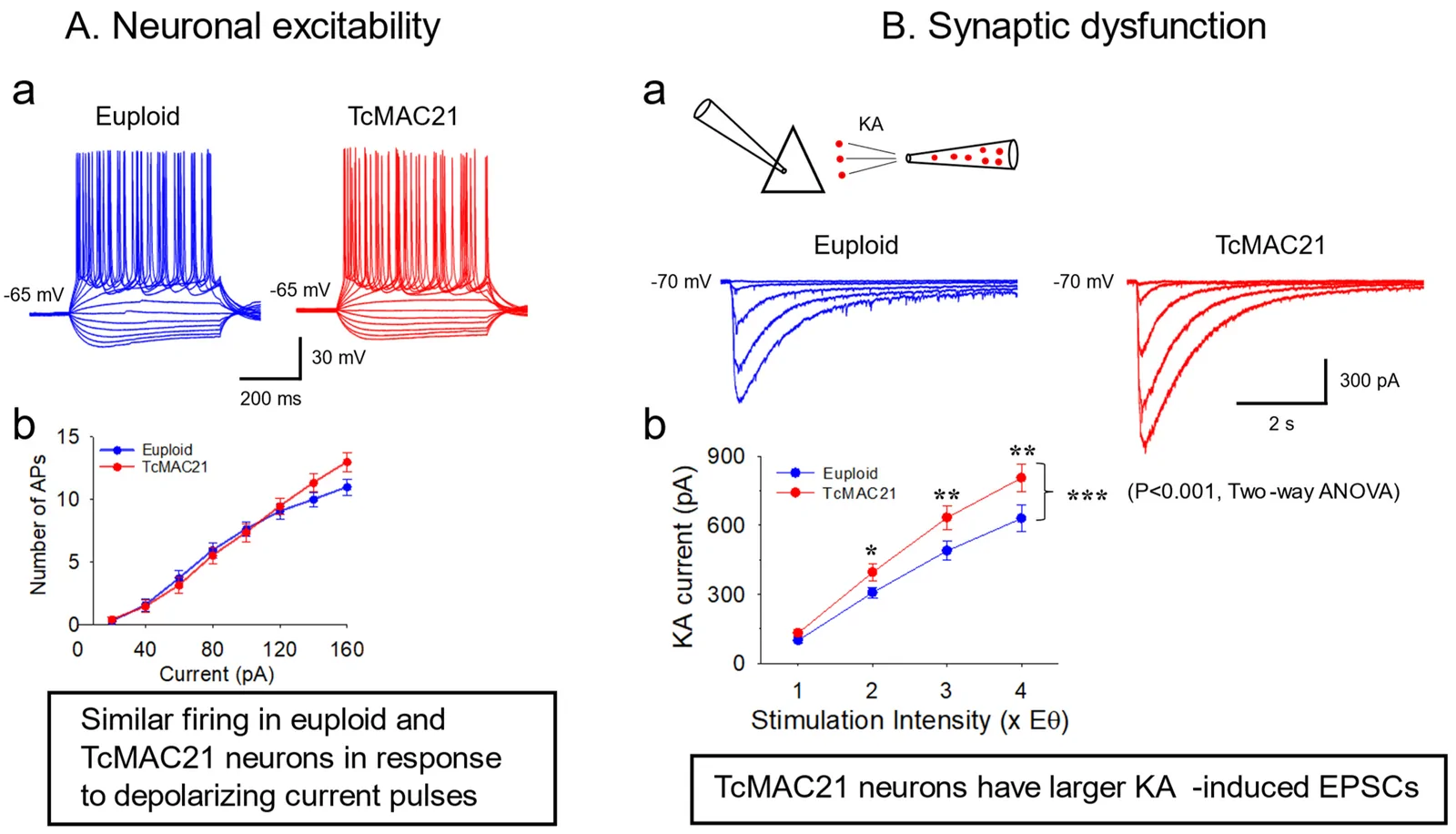
Neuronal excitability and synaptic dysfunction in layer V pyramidal neurons of euploid versus TcMAC21 mice. (**A**). (**a**). Intracellular recordings in response to hyperpolarizing and depolarizing current pulses show similar subthreshold voltage changes and firing rates in euploid (**left**) vs. TcMAC21 (**right**) neurons. (**A**). (**b**). Graph of input–output relationship showing similar number of action potentials elicited in both groups. With permission from Ref [85]. (**B**). (**a**). Experimental set-up with patch clamp recording from pyramidal neuron in response to various kainic acid puffs. Middle, excitatory postsynaptic currents from neurons of euploid mice (**left**) versus TcMAC21 mice (**right**). (**B**). (**b**). Graph showing significantly larger currents elicited by kainic acid in the TcMAC21 neurons. Asterisks denote level of significance. With permission from Ref [89]. Notes: mV, millivolts; ms, milliseconds; pA, picoamps; KA, kainic acid; TcMAC21, transchromosomic mouse artificial chromosome 21; APs, action potentials; Eθ, voltage threshold; ANOVA, analysis of variance.

**Figure 3:**
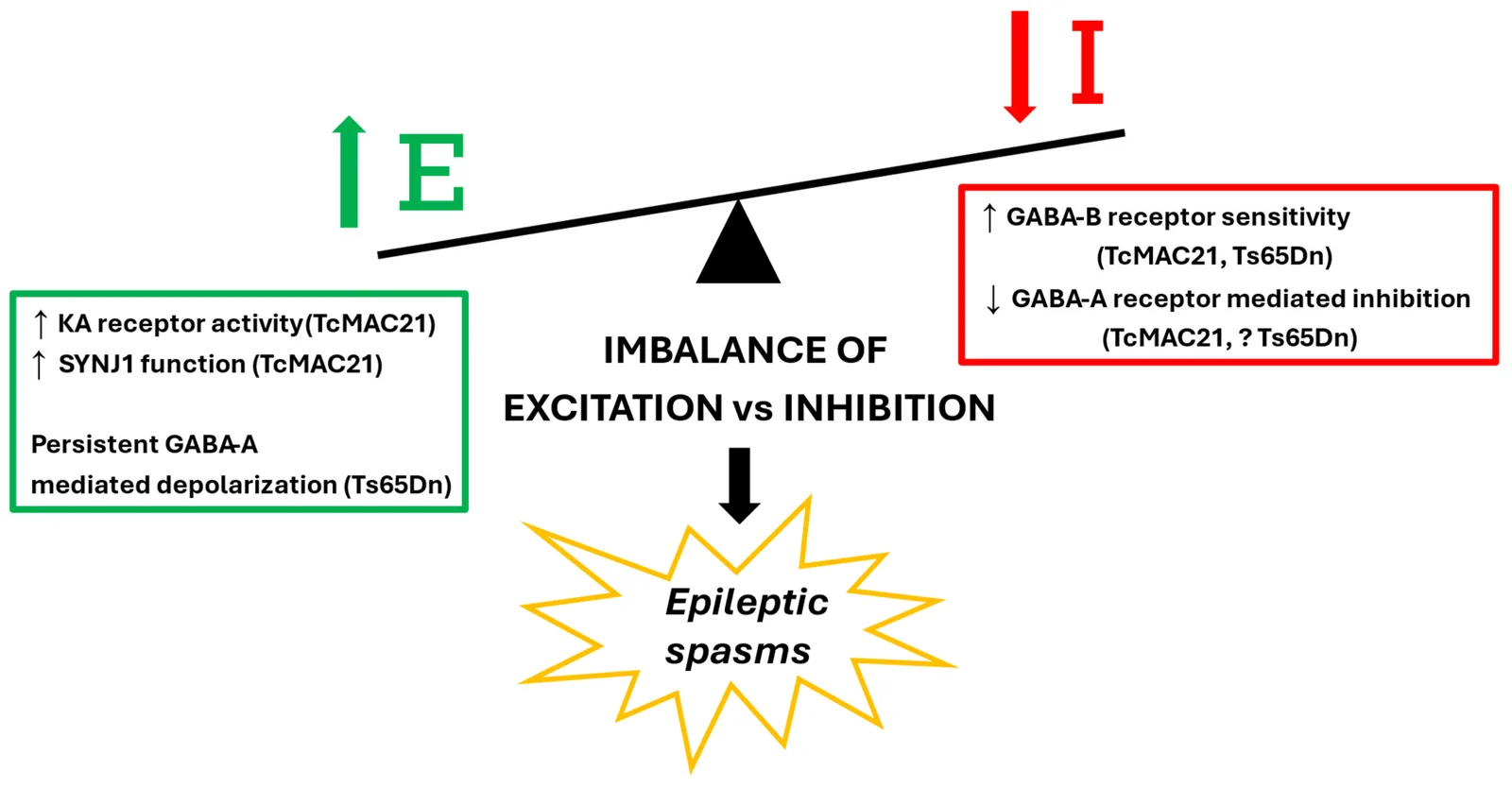
Cartoon illustrating the excitation/inhibition balance ratio in mouse models of Down syndrome (Ts65Dn, TcMAC21) and factors that increase excitation (green box), decrease inhibition (red box), or both, leading to epileptic spasms occurrence. Down syndrome mouse models in which data support the involvement of each factor are indicated in parentheses. Notes: GABA-A or GABA-B, gamma-aminobutyric acid receptors type A or B; SYNJ1, synaptojanin 1; E, excitation; I, inhibition.

**Figure 4:**
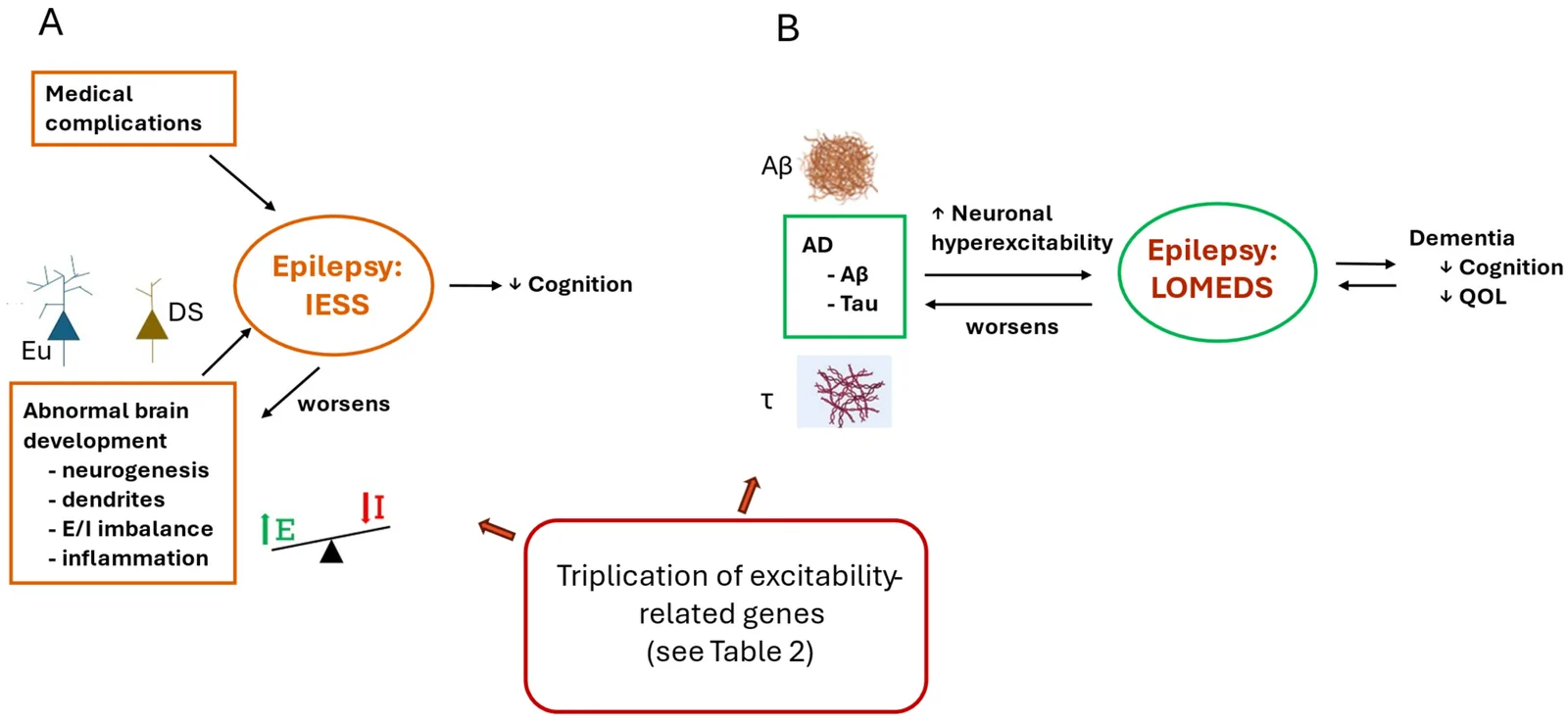
Relationship between pathophysiological etiologies, occurrence of epilepsy syndromes, and consequences in individuals with Down syndrome. (**A**) Under 1 year of age, IESS is the most frequent epilepsy syndrome, caused by abnormalities in brain development and medical complications of Down syndrome. (**B**) After age 30 years, myoclonic epilepsy emerges along with pathological (Aβ, tau) and clinical manifestations of Alzheimer’s disease. In both situations, the occurrence of seizures can worsen extant cognitive impairments. The relationship between epilepsy and dementia in Down syndrome is bidirectional. Inset cartoons illustrate simplified dendritic structure in trisomy 21 neurons (brown) compared with euploid neurons (blue), the imbalance between excitation (E) and inhibition (I), amyloid beta plaques; and τ neurofibrillary tangles. Notes: IESS, infantile epileptic spasms syndrome; E/I, excitation/inhibition; DS, Down syndrome; AD, Alzheimer disease; LOMEDS, late-onset myoclonic epilepsy in Down syndrome; QOL, quality of life; Eu, euploid.

**Table 1:** Epilepsy syndromes in Down syndrome.

	IESS in Down Syndrome	LOMEDS (PME-DS)
Age onset	4–10 mo	>30 yr
Prevalence	5–10% of infants with Down syndrome	At least 50% of Down syndrome adults exhibiting Alzheimer dementia; increase with age
Seizure type	Epileptic spasms	Myoclonic GTC Focal unaware
Electroencephalogram	Interictal–hypsarrhythmia Ictal-electrodecrement	Interictal–slow background Ictal–spike-waves
Structural/developmental abnormalities	Simplified dendritic tree Increased GABAergic interneurons	Plaques Tangles
Putative mechanisms	Increased GIRK2 Persistent GABAergic depolarization Excessive kainate receptor activity Reduced vesicle turnover	APP overexpression Aß-, tau-induced hyperexcitability Neurodegeneration
Associated signs	Cognitive plateau or regression	Cognitive deterioration
Treatment	Hormonal therapies (ACTH, oral corticosteroids), VGB	Broad spectrum ASM: LEV, VPA
Treatment response	Variable	Poor
Natural history/prognosis	Variable	Persistent functional decline

Notes: IESS, infantile epileptic spasms syndrome; LOMEDS, late-onset myoclonic epilepsy in Down syndrome; PME-DS, progressive myoclonic epilepsy in Down syndrome; GTC, generalized tonic-clonic; GABA, gamma-aminobutyric acid; GIRK2, G-protein inward rectifying potassium channel type 2; ACTH, adrenocorticotrophic hormone; APP, amyloid precursor protein; Aß, amyloid beta; ASM, antiseizure medication; LEV, levetiracetam; VPA, valproic acid, VGB, vigabatrin.

**Table 2:** Selected genes with potential role in neuronal excitability in Down syndrome.

Gene	Chromosome	Protein	Putative Mechanism
*KCNJ6*	21	GIRK2	Inward rectifying potassium channel
*GRIK1*	21	GluR5	Kainate-type glutamate receptor
*SYNJ1*	21	Synaptojanin type 1	Neurotransmitter vesicle recycling
*OLIG1* and *OLIG2*	21	Oligodendrocyte transcription factors	GABA interneuron generation, myelination
*APP*	21	ß-amyloid	Plaques–excitotoxicity, neurodegeneration
*MAPT*	17	Tau	Neurofibrillary tangles–excitotoxicity, neurodegeneration
*DYRK1A*	21	Tyrosine-phosphorylation-regulated kinase 1	RNA transcription, DNA repair
*DSCAM*	21	Down syndrome cell adhesion molecule	Dendrite branching
Other: *ETS2* *HMGN1* *RCAN1* *CCR3* *PSEN1*			Multiple functions in transcription/DNA repair, immune/inflammatory response, chromatin structure, calcium homeostasis, brain development, etc.–but no data on role in neuronal excitability or seizure predisposition

Notes: *KCNJ6*, potassium inwardly rectifying channel subfamily J member 6; GIRK2, G-protein inward rectifying potassium channel type 2; *GRIK1*, glutamate ionotropic receptor kainate type subunit 1; GluR5, glutamate ionotropic receptor kainate type subunit 1; *SYNJ1*, synaptojanin type 1, *OLIG1* and *2*, oligodendrocyte transcription factors 1 and 2; GABA, gamma-aminobutyric acid; *APP*, amyloid precursor protein; *MAPT*, microtubule associated protein tau; *DYRK1A*, dual specificity tyrosine phosphorylation regulated kinase 1A; *DSCAM*, Down syndrome cell adhesion molecule; *ETS2*, erythroblast transformation specific proto-oncogene 2 transcription factor; *HMGN1*, high mobility group nucleosome binding domain 1; *RCAN1* (DSCR1), regulator of calcineurin 1; *CCR3*, C-C motif chemokine receptor 3; *PSEN1*, presenilin 1.

## Data Availability

No new data were created or analyzed in this study. Data sharing is not applicable to this article.
